# Low genetic polymorphism of the PvMSP1-19 vaccine candidate among *Plasmodium vivax* isolates along the Thailand–Myanmar border

**DOI:** 10.1051/parasite/2026055

**Published:** 2026-09-28

**Authors:** Phiraya Pitchayatanakorn, Kanyarat Boonpeng, Natthakorn Inthanon, Chawaporn Khamkheaw, Suphattra Chanphetr, Janyaporn Tansuangkunnatham, Kaewkanha Kijprasong, Suttipat Srisutham

**Affiliations:** 1 Department of Clinical Microscopy, Faculty of Allied Health Sciences, Chulalongkorn University Bangkok 10330 Thailand; 2 Department of Parasitology, Faculty of Medicine, Chulalongkorn University Bangkok 10330 Thailand; 3 Lymphatic Filariasis and Tropical Medicine Research Unit, Chulalongkorn Medical Research Center, Faculty of Medicine, Chulalongkorn University Bangkok 10330 Thailand; 4 Phayabalsathanphrabaramee Hospital, Naung Proe Kanchanaburi Thailand

**Keywords:** *Plasmodium vivax*, PvMSP1-19, Genetic diversity, Vaccine candidate, Purifying selection, Thailand–Myanmar border

## Abstract

*Plasmodium vivax* remains the predominant malaria species in Thailand, accounting for approximately 97% of all reported cases, with transmission persisting in endemic border regions such as Kanchanaburi Province along the Thailand–Myanmar border. The C-terminal 19-kDa fragment of merozoite surface protein 1 (PvMSP1-19) has been proposed as a promising blood-stage vaccine candidate owing to its essential role in erythrocyte invasion and EGF-like domains. However, population-level genetic evidence from localized transmission foci in Southeast Asia remains limited. I this study, we investigated the genetic diversity and structural integrity of PvMSP1-19 in 109 *P. vivax* clinical isolates from Kanchanaburi, Thailand, between 2023 and 2025. Genomic DNA was extracted and the PvMSP1-19 region was amplified by PCR, sequenced by Sanger sequencing, and population genetic parameters were analyzed using DnaSP. Protein structure was predicted using AlphaFold3 and validated by MolProbity, PROCHECK, ProSA, and ERRAT2. Sequence analysis across 276 nucleotide positions identified only one segregating site, yielding two haplotypes with extremely low nucleotide and haplotype diversity. The dominant haplotype, corresponding to the Sal-1 reference strain, was present in 99.1% of isolates, and the single nucleotide substitution identified was synonymous, resulting in no amino acid substitution. A negative Tajima’s D value suggested a trend consistent with purifying selection, although this was not statistically significant. Structural modelling of PvMSP1-19 confirmed a stable, high-quality conformation. These findings demonstrate that PvMSP1-19 is highly conserved in *P. vivax* populations circulating along the Thailand–Myanmar border and support its potential as a promising antigen for blood-stage vaccine development targeting vivax malaria in Southeast Asia.

## Abstract (fr)

*Plasmodium vivax* demeure l’espèce prédominante responsable du paludisme en Thaïlande, représentant environ 97 % de tous les cas signalés, et sa transmission persiste dans les régions frontalières endémiques, telles que la province de Kanchanaburi, située le long de la frontière entre la Thaïlande et le Myanmar. Le fragment C-terminal de 19 kDa de la protéine de surface 1 du mérozoïte (PvMSP1-19) a été proposé comme candidat vaccin prometteur contre le stade sanguin du parasite, en raison de son rôle essentiel dans l’invasion des érythrocytes et de la présence de domaines de type EGF. Toutefois, les données génétiques à l’échelle de la population, issues de foyers de transmission localisés en Asie du Sud-Est, restent limitées. Cette étude a examiné la diversité génétique et l’intégrité structurelle de la protéine PvMSP1-19 chez 109 isolats cliniques de *P. vivax* provenant de Kanchanaburi (Thaïlande) collectés entre 2023 et 2025. L’ADN génomique a été extrait et la région codant pour PvMSP1-19 a été amplifiée par PCR puis séquencée par la méthode de Sanger, et les paramètres de génétique des populations ont été analysés à l’aide du logiciel DnaSP. La structure protéique a été prédite par AlphaFold3 et validée par les outils MolProbity, PROCHECK, ProSA et ERRAT2. L’analyse des séquences sur 276 positions nucléotidiques a révélé un seul site polymorphe, donnant lieu à deux haplotypes caractérisés par une diversité nucléotidique et une diversité haplotypique extrêmement faibles. L’haplotype dominant, correspondant à la souche de référence Sal-1, était présent chez 99,1 % des isolats, et la substitution nucléotidique unique identifiée était synonyme, n’entraînant aucune modification de la séquence en acides aminés. Une valeur négative du test D de Tajima suggérait une tendance compatible avec une sélection purificatrice, bien que ce résultat ne fût pas statistiquement significatif. La modélisation structurelle de PvMSP1-19 a confirmé une conformation stable et de haute qualité. Ces résultats démontrent que la protéine PvMSP1-19 est hautement conservée au sein des populations de *P. vivax* circulant le long de la frontière entre la Thaïlande et le Myanmar, et confortent également son potentiel en tant qu’antigène prometteur pour le développement d’un vaccin ciblant le stade sanguin du paludisme à *P. vivax* en Asie du Sud-Est.

## Introduction

Malaria continues to impose a substantial global health burden, disproportionately affecting populations in tropical and subtropical regions where transmission by infected female *Anopheles* mosquitoes is endemic. The causal agents of malaria belong to the genus *Plasmodium*, with five species known to infect humans. According to the World Health Organization (WHO) World Malaria Report 2024, an estimated 263 million malaria cases and 597,000 deaths were recorded globally in 2023, representing an increase of approximately 11 million cases compared with the preceding year, reflecting the persistence of the disease despite intensive international control efforts [28].

Among the five *Plasmodium* species infecting humans, *Plasmodium vivax* is distinguished by its exceptionally broad geographic distribution and constitutes the predominant cause of malaria morbidity outside sub-Saharan Africa, particularly across Asia, Latin America, and the Western Pacific [3]. In Thailand, *P. vivax* accounted for approximately 97% of all reported malaria cases in 2023, with transmission persisting predominantly in border regions characterized by high population mobility, including Kanchanaburi Province along the Thailand–Myanmar border [23, 28].

Two biologically distinctive features render *P. vivax* particularly challenging from a public health perspective. First, the parasite is capable of forming dormant liver-stage forms, termed hypnozoites, which can persist in hepatocytes for weeks to months or even years following primary infection. Upon activation, hypnozoites initiate secondary blood-stage infection – termed relapse – thereby perpetuating transmission and complicating attempts at malaria eradication [10, 18]. Second, *P. vivax* preferentially develops in reticulocytes, a restriction that severely limits the establishment of continuous *in vitro* culture systems and has substantially hindered experimental and vaccine-related research [26]. In Thailand, sustained reductions in malaria incidence over the past two decades have been documented. However, transmission has not been eliminated and remains concentrated in border regions, where *P. vivax* has become the predominant species and continuous surveillance is therefore necessary for monitoring the development of drug resistance. The situation has been exacerbated by migration of individuals from Myanmar and by the spread of parasites through mosquitoes in forested border regions, with malaria cases increasing markedly in 2023, the majority of which were attributed to *P. vivax* infections [23]. On the Thai–Myanmar border, *P. vivax* cases are significantly associated with local mosquito capture rates and are less directly linked to migrant status than *P. falciparum*, indicating sustained local transmission that underlines the different timelines and requirements for elimination of the two species in the same region [24].

The development of an effective vaccine against *P. vivax* is widely regarded as integral to long-term malaria control and elimination strategies. However, no licensed vaccine currently exists for this species, in contrast to the two WHO-prequalified vaccines targeting *P. falciparum, which are* RTS, S/AS01 (prequalified July 2022) and R21/Matrix-M (prequalified December 2023) [2]. This gap is largely attributable to the parasite’s complex life cycle, reticulocyte tropism, and extensive antigenic diversity across blood-stage antigens, all of which complicate the development of broadly protective immunity [9].

Among blood-stage vaccine candidates, merozoite surface protein 1 (MSP1) has been extensively investigated owing to its essential role in erythrocyte invasion. The PvMSP1 gene is encoded on chromosome 7 and is organized into 13 alternating conserved and polymorphic blocks. During merozoite maturation, MSP1 undergoes sequential proteolytic processing, yielding a series of fragments including a 42-kDa C-terminal product that is subsequently cleaved into 33-kDa and 19-kDa subfragments [12]. The 19-kDa C-terminal fragment (PvMSP1-19), corresponding to block 13, harbors two disulfide-stabilized epidermal growth factor (EGF)-like domains essential for receptor interaction during erythrocyte invasion, and remains anchored on the merozoite surface throughout the invasion process [4].

Global analyses have demonstrated that the PvMSP1-19 region exhibits highly conserved sequences across geographically diverse parasite populations spanning Asia, South America, North America, and Oceania, with markedly lower nucleotide diversity than co-circulating blood-stage antigens such as PvAMA1, supporting its candidacy for a broadly protective multivalent vaccine formulation [13]. The structural constraints imposed by the disulfide-bonded EGF-like domains are considered the principal basis for this conservation, as amino acid substitutions in this region are likely to disrupt receptor-binding function and reduce parasite fitness [4, 12]. Nevertheless, low-frequency polymorphisms within PvMSP1-19 have been reported in several endemic settings and may have implications for antibody recognition and vaccine efficacy [20].

In Thailand, the genetic diversity of *P. vivax* populations has been characterized across multiple antigenic loci; however, population-level data specifically addressing PvMSP1-19 conservation in localized endemic foci along the Thailand–Myanmar border, particularly in Kanchanaburi Province, remain limited. Such fine-scale characterization of antigen diversity in geographically distinct transmission foci is essential for evaluating the anticipated breadth of protection conferred by candidate vaccines in endemic populations. A previous study from the same overarching project investigated molecular markers associated with *P. vivax* drug resistance in Kanchanaburi Province [6]. Although 31 isolates overlapped between the previous and present studies, the present study investigated a distinct molecular target, the PvMSP1-19 vaccine-candidate region, and generated distinct sequence data and analyses. Therefore, the present study investigated the genetic diversity, haplotype structure, and signatures of natural selection of PvMSP1-19 in 109 clinical *P. vivax* isolates from Kanchanaburi Province, western Thailand, with the aim of providing population-specific evidence to inform blood-stage vaccine development targeting vivax malaria in Southeast Asia.

## Methods

### Ethics approval

The study protocol was reviewed and approved by the Ethics Review Committee for Research Involving Human Subjects, Health Science Group, Chulalongkorn University, Thailand (COA No. 250/66). Subsequent approvals were obtained for continuation of the study activities in 2024 (COA No. 254/67) and 2025 (COA No. 328/68). Prior to enrollment, participants were informed about the study’s objectives, confidentiality measures, and their right to withdraw at any time. Written informed consent was obtained from all participants. This study was conducted as part of an overarching project investigating the molecular epidemiology and genetic characteristics of *P. vivax*. The project also supported a previously published investigation of *P. vivax* drug-resistance markers [6]; however, the present study addressed a distinct molecular target and research question.

### Sample collection and DNA extraction

A total of 143 *P. vivax*–positive EDTA whole-blood samples were collected from patients with malaria attending Phayabalsathanphrabaramee Hospital, Naung Proe, Kanchanaburi Province, Thailand, between 2023 and 2025. Blood samples were collected before the initiation of antimalarial treatment and stored at 4 °C. Genomic DNA was extracted from whole blood samples within 3 days of collection using a GF-1 Blood DNA Extraction Kit (Vivantis, Selangor, Malaysia), according to the manufacturer’s instructions. Of the 143 samples, 114 yielded successful PCR amplification of the *PvMSP1-19* fragment. Sequencing data of acceptable quality were obtained from 109 samples, which were included in the final sequence polymorphism and genetic diversity analyses. Of these 109 isolates, 31 overlapped with samples included in a previous study of *P. vivax* drug-resistance markers [6]. The present study examined the distinct PvMSP1-19 target, and the PCR products, sequence data, analyses, and results reported here have not been published previously.

### PCR amplification and sequencing

PCR reactions were performed using 0.25 μM of each primer, 0.2 mM dNTPs, 1.2 mM MgCl_2_, and 2 U of Taq DNA polymerase (Vivantis). The amplification conditions were as follows: initial denaturation at 95 °C for 5 min; 40 cycles of denaturation at 94 °C for 1 min, annealing at 60 °C for 30 s, and extension at 72 °C for 1 min; followed by a final extension at 72 °C for 7 min. Amplicons were analyzed by 1.5% agarose gel electrophoresis, purified, and subjected to single-pass Sanger sequencing using the reverse primer by Macrogen Inc. (Seoul, Republic of Korea). Chromatograms were visually inspected, and low-quality sequence regions were trimmed before alignment and subsequent analyses.

The PvMSP1-19 gene was amplified using specific forward (5′–ATGAGCTCCGAGCACACA–3′) and reverse (5′–TTAAAGCTCCATGCACAGGA–3′) primers [5]. PCR reactions were performed using 0.25 μM of each primer, 0.2 mM dNTPs, 1.2 mM MgCl_2_, 2 U Taq DNA polymerase (Vivantis). The PCR amplification was performed under the following conditions: (1) pre-denaturation at 95 °C for 5 min; (2) 40 cycles of denaturation at 94 °C for 1 min, annealing at 60 °C for 30 s, and extension at 72 °C for 1 min; (3) post extension at 72 °C for 7 min. PCR products were subsequently analyzed by 1.5% agarose gel electrophoresis. PCR products were purified and subjected to single-pass Sanger sequencing using the reverse primer by Macrogen Inc. The chromatograms were visually inspected, and low-quality sequences were trimmed before alignment and subsequent analyses.

### Sequence alignment and polymorphism analysis

The sequences were aligned using Clustal Omega [17] and analyzed with reference to the *P. vivax* Sal-1 reference strain MSP1 sequence (Accession number: NC_009912.1; positions 1,162,662–1,162,997). A phylogenetic tree was constructed using MEGA 12 [14]. Sequence polymorphism and genetic diversity parameters, including number of segregating sites (S), haplotype diversity (Hd), nucleotide diversity (*π*), and Tajima’s D, were calculated using DnaSP software [21].

### Structural prediction and validation

A three-dimensional structural model of PvMSP1-19 was generated using AlphaFold3 [1] and visualized in PyMOL [22]. Model quality was assessed using MolProbity [8], PROCHECK [15], ProSA [27], and ERRAT2 [7]. Secondary structure alignment was generated using ESPript [11].

## Results

### Sequence conservation and polymorphism

Multiple sequence alignment of all 109 isolates and the PvMSP1-19 (NC_009912.1:1162662-1162997) revealed only one polymorphic site across the entire 276-bp PvMSP1-19 region, yielding two distinct haplotypes (Table 1). A synonymous substitution (C5187T) was detected in the MP0406 isolate, with no effect on the amino acid sequence. (Figs. 1 and 2). Consistently, phylogenetic analysis demonstrated extremely low genetic divergence, with all isolates clustering tightly, while MP0406 formed a slightly distinct branch due to this single nucleotide variation (Fig. 3). Therefore, the high level of sequence conservation relative to the reference strain (Sal-1) indicates that PvMSP1-19 is a highly conserved genomic region, supporting its potential as a promising vaccine candidate for *P. vivax*.

**Table 1:** Genetic diversity parameters of PvMSP1-19 gene for vaccine target evaluation.

Parameter	Value
Number of sequences (*n*)	109
Number of sites	276
Segregating sites (S)	1
Number of haplotypes (h)	2
Haplotype diversity (Hd)	0.018 ± 0.018
Nucleotide diversity (*π*)	0.00007
Average nucleotide differences (*k*)	0.018
Tajima’s D	−1.01826

**Figure 1:**
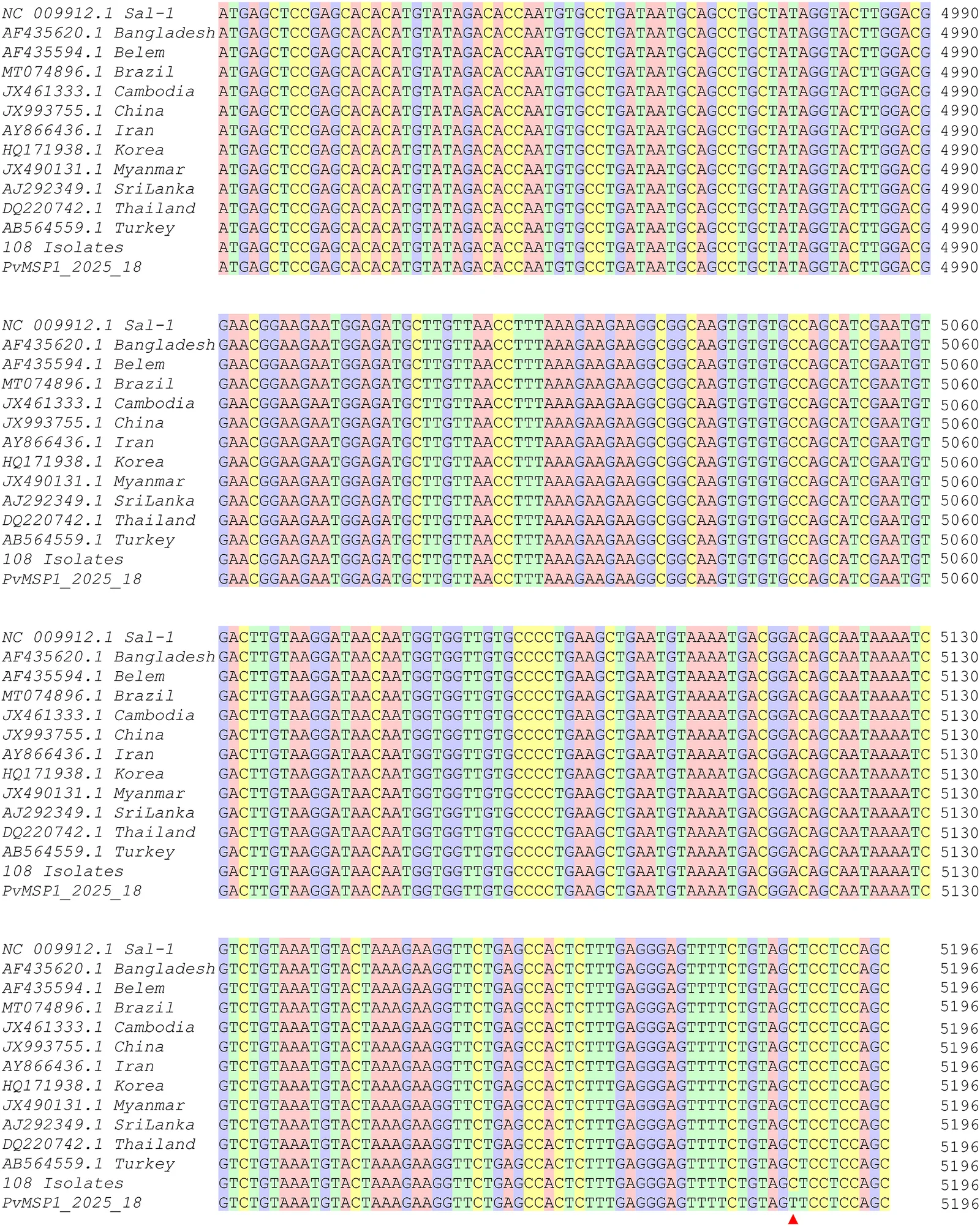
Multiple sequence alignment of PvMSP1-19 nucleotide sequences from different geographic regions and 109 isolates from this study. The mutation site is marked with a red arrow under the sequences. The alignment was generated with CLUSTAL Omega and visualized by Jalview.

**Figure 2:**
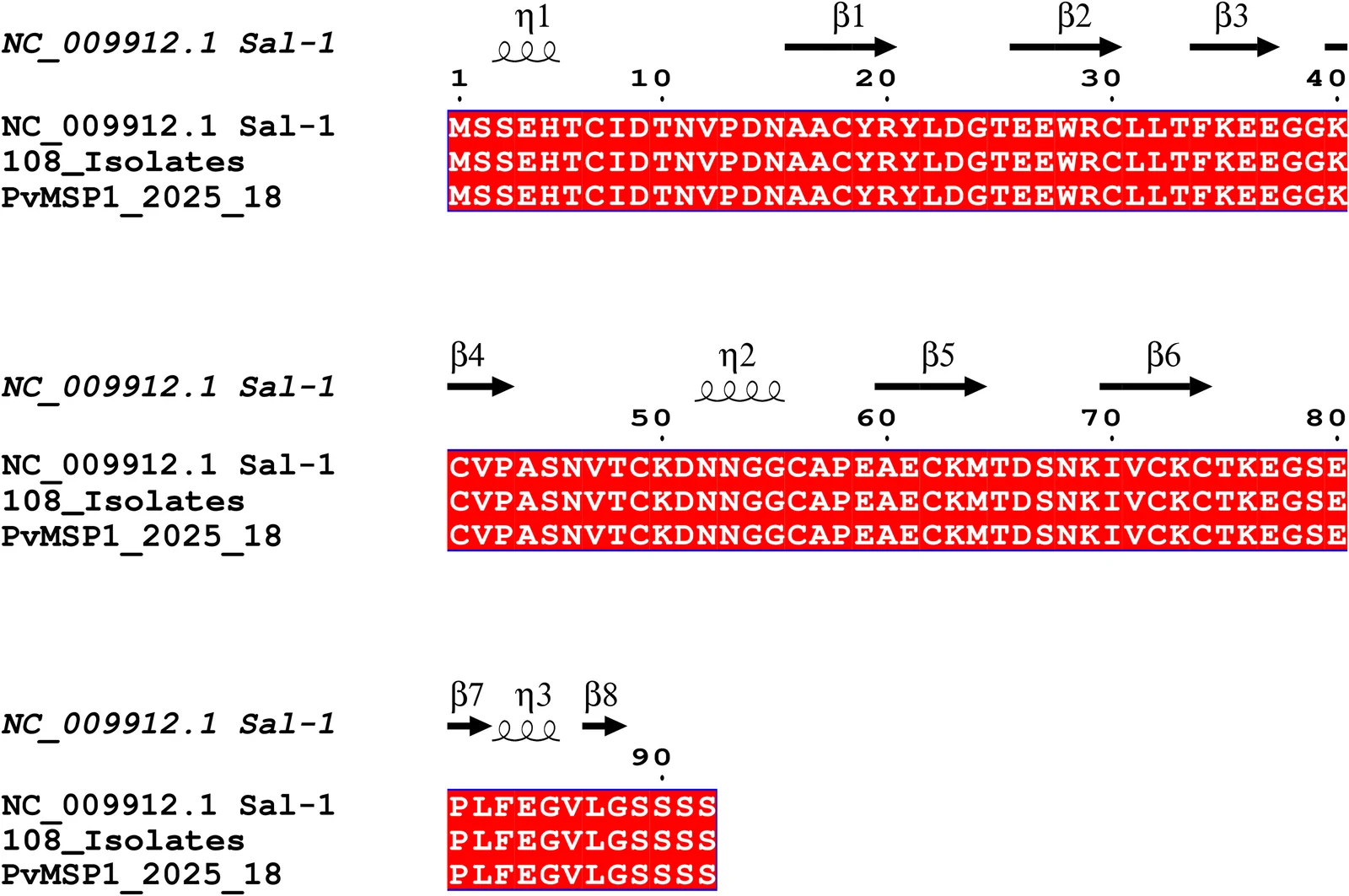
Secondary structure alignment of the PvMSP1-19. The alignment was generated by using Clustal Omega, and the secondary structures of the PvMSP1-19 were aligned by using the ESpript program.

**Figure 3:**
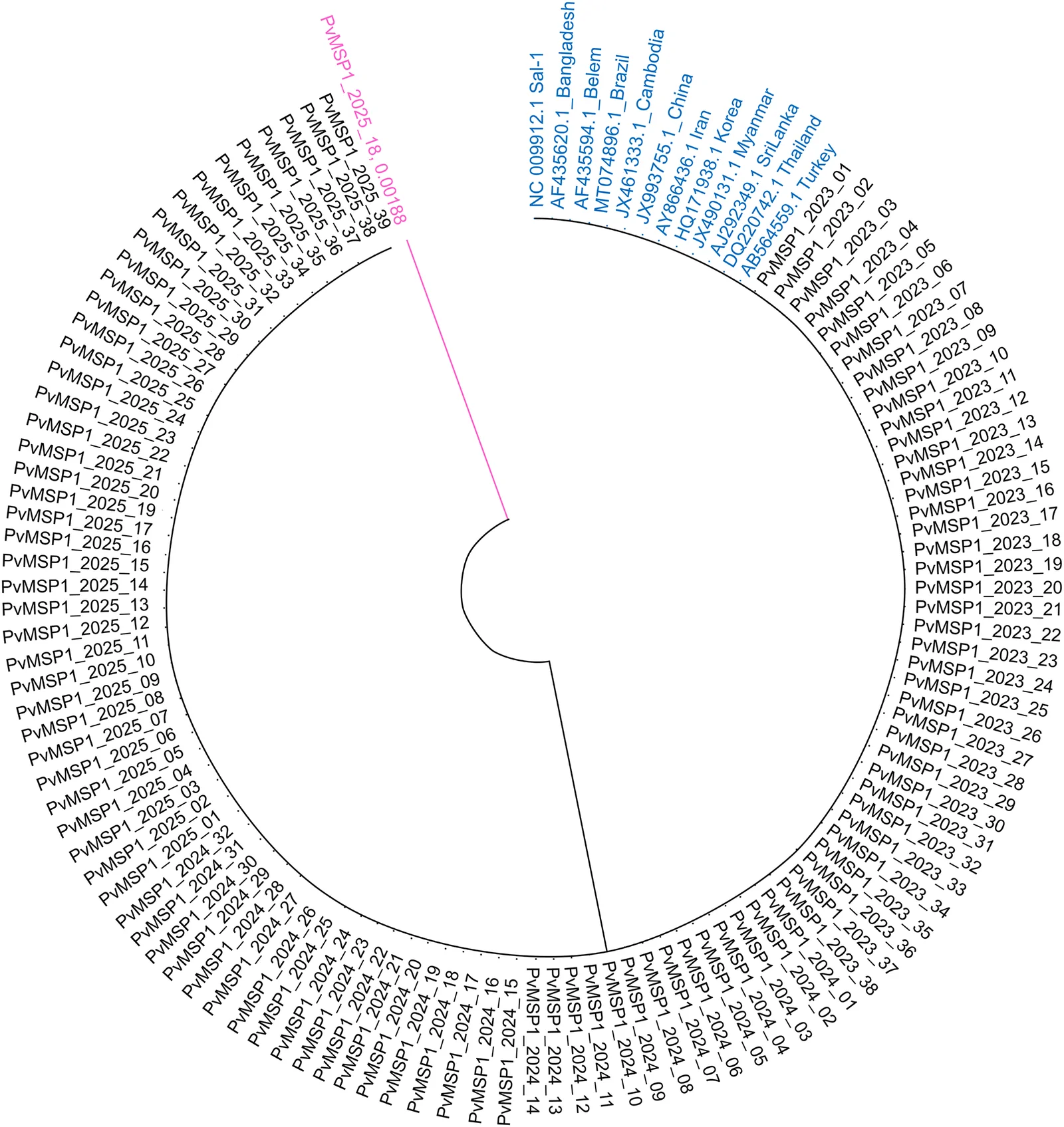
Phylogenetic tree of PvMSP1-19 sequences constructed using the Maximum Likelihood method based on the Tamura–Nei model. The tree with the highest log likelihood (−384.51) is shown. Bootstrap values (1,000 replicates) are indicated at the nodes. The analysis included 121 nucleotide sequences with 276 aligned positions. The reference strain (Sal-1) and sequences from different regions are shown in blue, while the isolate MP0406 is shown in pink. Branch lengths are not shown for identical isolates; only MP0406 is displayed, showing a slightly longer branch due to a single nucleotide difference.

### Genetic diversity analysis

Population genetic analysis demonstrated extremely low genetic variability within the Kanchanaburi *P. vivax* population (Table 1). One segregating site was identified, yielding two haplotypes with a haplotype diversity (Hd) of 0.018 ± 0.018. The nucleotide diversity (π) was 0.00007, and the average number of nucleotide differences (*k*) was 0.018. The dominant haplotype (H1), corresponding to the Sal-1 reference type, was observed in 108 of 109 isolates (99.1%). The minor haplotype (H2), defined by a single C5187T substitution, was detected in only one isolate (0.9%). Tajima’s D value was negative (−1.01826) but not statistically significant (*p* > 0.10), suggesting no strong deviation from neutral evolution in the Kanchanaburi parasite population.

### Structural analysis

The predicted structure of PvMSP1-19 exhibited well-defined secondary structural elements, including α-helices and β-strands, consistent with a stable and folded protein conformation (Fig. 4). Structural validation confirmed high model quality (Table 2), with a MolProbity score of 1.31, 92.9% of residues in favored regions of the Ramachandran plot, and an ERRAT2 score of 95.7, supporting the reliable structural model. The structural stability of PvMSP1-19 further supports its potential as a conserved and functionally important vaccine target.

**Figure 4:**
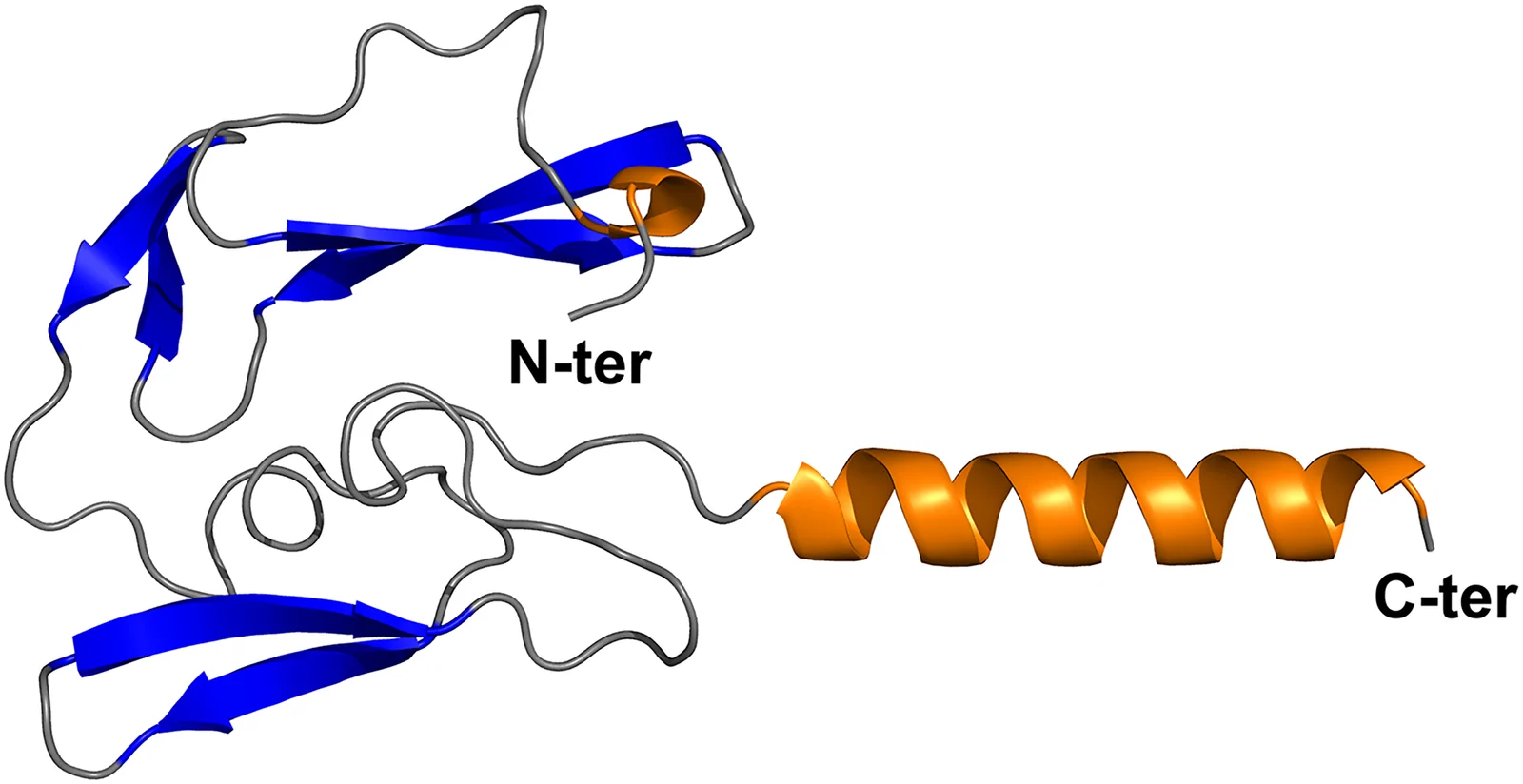
Model structure of PvMSP1-19 generated using AlphaFold3 and visualized in PyMOL. Beta-strands are shown as blue arrows and alpha-helices as orange spirals, and loops in grey.

**Table 2:** Quality of the PvMSP1-19 model evaluated by the MolProbity, PROCHECK, ProSA, and Errat2 programs.

Evaluation program	PvMSP1-19 model	Criteria for reliable model
MolProbity		
MolProbity score	1.31	< 2.0
Clash score	1.81	
PROCHECK		
Ramachandran plot		
Residues in most favored regions (%)	92.9	
Residues in additional allowed regions (%)	7.1	< 2% of residues in disallowed region
Residues in generously allowed regions (%)	0.0	
Residues in disallowed regions (%)	0.0	
Overall G-factor	−0.12	Close to zero
ProSA *z*-score	−4.7	Within an acceptable range of *z*-score plot
Errat2	95.70	> 95%

## Discussion

In this study, we characterized the genetic diversity of PvMSP1-19 in 109 *P. vivax* clinical isolates from Kanchanaburi Province along the Thailand–Myanmar border, one of the principal endemic foci of vivax malaria in Southeast Asia [23]. PvMSP1-19 was found to be virtually monomorphic, with only a single synonymous substitution detected across 276 bp, consistent with a trend toward purifying selection.

The extremely low nucleotide diversity (π = 0.00007) and minimal haplotype diversity (Hd = 0.018) observed in the current study are consistent with the global pattern of PvMSP1-19 conservation. A multi-country analysis encompassing 682 sequences identified only 14 haplotypes with an overall π = 0.00937 and Hd = 0.20, with Thailand-derived populations exhibiting some of the lowest nucleotide divergence values globally [13]. The present Kanchanaburi cohort falls well within, and indeed below, this range, indicating that the high conservation previously reported at the regional level is maintained at the scale of a localized transmission focus. This stands in marked contrast to the 42-kDa region of PvMSP1, which exhibited nucleotide diversity as high as π = 0.027 and haplotype diversity of Hd = 0.962 in Thai border populations [25], underscoring that the conservation of PvMSP1-19 is a specific property of this C-terminal domain rather than a general characteristic of the PvMSP1 locus.

The structural validation performed in this study provides a mechanistic explanation for this pattern. The AlphaFold3-predicted model achieved high scores across all quality metrics, consistent with a well-folded, functional protein. The two disulfide-stabilized EGF-like domains impose strong constraints on sequence variation, as substitutions disrupting these bonds or the domain fold are expected to be highly deleterious to parasite fitness, by analogy with the well-characterized *P. falciparum* MSP1-19 [4, 12]. The synonymous C5187T substitution identified, which does not alter the encoded amino acid, is consistent with purifying selection operating at this locus, as supported by the negative Tajima’s D value (−1.01826), although this did not reach statistical significance (*p* > 0.10), precluding a definitive conclusion regarding the selective regime from this dataset alone.

These findings carry direct implications for vaccine development. The near-universal prevalence of the H1 haplotype (99.1%) indicates that a Sal-1-based formulation would be antigenically representative of essentially all circulating parasites in this population. Since both haplotypes encode identical proteins, no amino acid diversity exists to drive immune evasion. Naturally acquired IgG1 and IgG3 responses to PvMSP1-19 have been documented in 55% of *P. vivax*-exposed individuals along this border, with antibody levels inversely associated with parasite density [19], further supporting the immunogenic potential of this antigen in the target population. Collectively, the sequence conservation, structural integrity, and natural immunogenicity of PvMSP1-19 support its prioritization as a vaccine candidate for blood-stage *P. vivax* in Southeast Asia [13, 16]. Nonetheless, future studies employing amplicon deep sequencing and longitudinal sampling across multiple transmission seasons are warranted to more comprehensively characterize population-level diversity at this locus.

This study has several limitations. First, all samples were collected from a single study location in Kanchanaburi Province; therefore, the findings may not fully represent the genetic diversity of *PvMSP1-19* in other areas of Thailand or Southeast Asia. Second, sequencing was performed in a single direction using the reverse primer. Although chromatograms were inspected and low-quality regions were trimmed, sequencing errors or ambiguous nucleotide calls cannot be completely excluded. Third, 31 of the 109 isolates overlapped with a previously published study of *P. vivax* drug-resistance markers [6]. However, the present study addressed a distinct vaccine-candidate target, and the *PvMSP1-19* sequence data, analyses, and findings have not been reported previously. Future studies should include samples from multiple endemic areas and use bidirectional sequencing or amplicon deep sequencing to confirm rare variants and further characterize population-level diversity.

## Conclusion

The PvMSP1-19 region of *P. vivax* is highly conserved in clinical isolates from Kanchanaburi Province, Thailand, with near-universal prevalence of the Sal-1 reference haplotype and only a single synonymous polymorphism across 109 isolates. Evidence of purifying selection and structural validation of the predicted protein model further support the functional constraint acting on this locus. These findings should be interpreted in light of the single-site sampling design and the use of single-direction sequencing. Collectively, these findings provide population-specific evidence that PvMSP1-19 constitutes a broadly representative and structurally stable target for blood-stage vaccine development against *P. vivax* in endemic border regions of Southeast Asia.

## Data Availability

The datasets used during this study are available from the corresponding author on reasonable request.
